# Adrenaline intravenous therapy persistence grade I severe allergic reaction: A case report and literature review

**DOI:** 10.1097/MD.0000000000039193

**Published:** 2024-08-02

**Authors:** Wei Wang, Ying Zhang, Li Tao, Tong-Xue Jiang, Ning Wang, Wen-Hui Zhai

**Affiliations:** a Emergency Department, 305 Hospital of PLA, Beijing, China; b Outpatient Department, 305 Hospital of PLA, Beijing, China.

**Keywords:** abdominal pain, allergic reactions, case report, epinephrine, rash

## Abstract

**Rationale:**

At present, there is still insufficient understanding of the progression from persistent allergic reactions to severe reactions. Adrenaline remains the preferred medication for severe allergic reactions, and intramuscular injection of adrenaline can also be considered for patients with grade I reactions that are difficult to alleviate gastrointestinal symptoms. It is worth further discussing whether it is possible to break the conventional intramuscular injection recommended by the guidelines when the effect of intramuscular injection is not ideal for persistent grade I severe allergic reactions.

**Patient concerns:**

A young male, 20 years of age, was admitted to emergency department because of repeated rash for 3 days and abdominal pain for 6 hours after taking traditional Chinese medicine. After hormone therapy, the rash continued to recur and secondary gastrointestinal symptoms occurred on the 3th day. Adrenaline intramuscular injection was given to temporarily relieve the rash and abdominal pain, but symptoms still persisted.

**Diagnosis:**

The patient was diagnosed with persistent severe allergic reaction (grade I).

**Interventions:**

Continuous intravenous infusion of low-dose adrenaline under electrocardiographic monitoring, real-time monitoring of heart rate and blood pressure, and routine treatment with methylprednisolone, diphenhydramine, calcium gluconate, and cetirizine. During this period, adrenaline intramuscular injection is temporarily added when abdominal pain symptoms are obvious. The entire treatment process used a total of 6.8 mg of adrenaline.

**Outcomes:**

During the entire period of adrenaline intervention, the patient did not experience any new discomfort, and there were no abnormal fluctuations in heart rate, rhythm, or blood pressure. The symptoms of rash and abdominal pain gradually improved.

**Lessons:**

For patients with persistent grade I severe allergic reactions, intravenous administration of low-dose adrenaline under close vital sign monitoring is safe, feasible, and highly effective in preventing biphasic, persistent, or worsening allergic reactions.

## 1. Introduction

Severe allergic reactions refer to systemic hypersensitivity reactions that occur suddenly, rapidly, and can be fatal after exposure to allergens.^[1]^ Most of these reactions occur rapidly, gradually worsen, and then completely disappear, especially after proper treatment. The symptoms of a very small number of patients can last for several days or weeks, known as persistent severe allergic reactions.^[2]^ In such severe allergic reactions, the reasonable use of adrenaline is extremely important. For persistent grade I severe allergic reactions, whether adrenaline should be administered intramuscularly according to guidelines or intravenously based on clinical characteristics is worth discussing. Discussion on the rationality of intravenous use of adrenaline in the treatment of a case of persistent severe allergic reaction caused by traditional Chinese medicine decoction in our hospital.

## 2. Case presentation

A 20-year-old male presented to the emergency department on March 1, 2023 with recurrent rash all over the body for 3 days and abdominal pain for 6 hours. The patient developed a rash all over the body with itching after taking traditional Chinese medicine decoction for insomnia on February 26, and was treated in the health room with promethazine 25 mg intramuscularly and loratadine 10 mg orally, and the rash temporarily subsided, but the rash continued to recur. On February 28, he presented to the dermatology department of our hospital and was diagnosed with allergic dermatitis, and the rash did not improve after oral treatment with dexamethasone 10 mg and cetirizine 10 mg. 6 hours prior to medical attention, there was a sudden onset of periumbilical colic (numeric rating scales [NRS]: 5–6) during sleep, which was paroxysmal and aggravated, accompanied by watery stools, about 6 to 7 times, accompanied by nausea and vomiting 3 times, no vomiting of blood, no mucus or purulent stools. During the course of the disease, there was no discomfort or shortness of breath in the throat, no dizziness, fatigue, blackness, fever or other symptoms.

He was in good health in the past, and he reported that he had been allergic to seafood and cosmetics by allergen testing, and denied a history of drug allergies. At the time of presentation, the body temperature was 36.8 °C, the heart rate was 79 beats/min, the blood pressure was 140/90 mm Hg, the breath was 16 breaths/min, the oxygen saturation of the transcutaneous fingerstick was 98% (without oxygen), and the body weight was 65 kg. On examination, there was a generalized erythematous and hyperemic maculopapular rash with mild pruritus, which was pleomorphic and some may be joined into patches. There was no hyperemia and edema in the pharynx, and the breath sounds in both lungs were clear. The abdomen was flat and soft, bowel sounds were about 3 times/min, epigastric and periumbilical tenderness, and there was no rebound tenderness.

This patient experienced persistent abdominal pain accompanied by nausea and vomiting during the treatment of allergic rash, lasting for 5 days, and other causes of abdominal pain were excluded. Based on clinical characteristics and the recommendations of the “Emergency Guidelines for Severe Allergic Reactions” written by Xiao-Tong Li et al, a preliminary diagnosis of persistent severe allergic reactions was made (grade I). Immediately monitored the electrocardiogram and administered methylprednisolone 40 mg intravenous infusion, promethazine 25 mg intramuscular injection, 10% glucose injection 20 mL and calcium gluconate injection 1 g intravenous injection, and cetirizine 10 mg oral treatment. The patient’s rash did not improve, and abdominal pain worsened compared to before (NRS: 6–7). Immediately administered epinephrine 0.3 mg intramuscular injection, 10 minutes after intramuscular injection, the patient’s abdominal pain improved compared to before (NRS: 1–2), and the rash around the body was decreased compared to before.

On the same night, the patient experienced abdominal pain, vomiting, and diarrhea again after eating a small amount of bread, and the rash all over the body worsened again, with obvious congestion, protruding from the skin surface, connected in patches, and a noticeable itching sensation. Immediate intramuscular injection of 10 mg of scopolamine and 0.5 mg of adrenaline was given, and the abdominal pain and rash were relieved again. Subsequently, a small dose of epinephrine (speed was 0.02 μg/kg min) was administered under electrocardiogram monitoring, and the heart rate and blood pressure were recorded every hour. Routine treatment was performed with methylprednisolone 40 mg intravenous infusion 1/day, diphenhydramine 20 mg intramuscular injection 1/day, 10% glucose injection 20 mL and calcium gluconate injection 1 g intravenous infusion 1/day, cetirizine 10mg oral administration 1/day, and loratadine 10 mg oral administration 1/night. During this period, epinephrine intramuscular injection with a dose of 0.5 mg each time was temporarily added when abdominal pain symptoms occurred.

**Figure 1.**
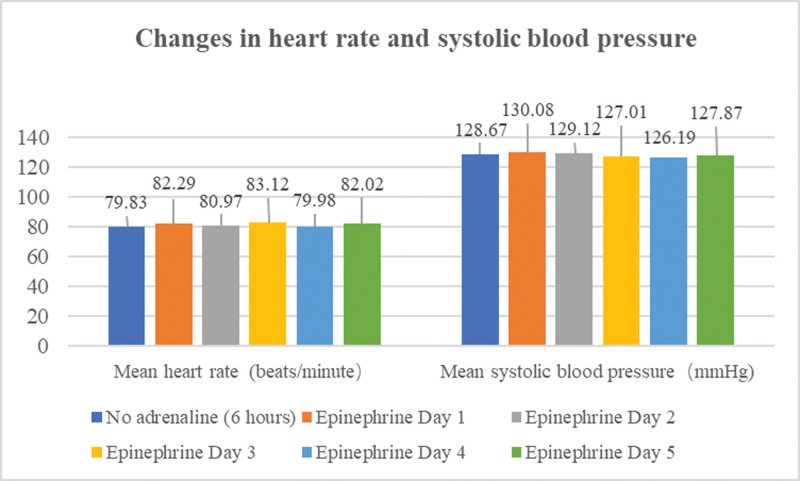
Changes in heart rate and systolic blood pressure before and after intravenous epinephrine pumping (there was no statistically significant difference in heart rate and systolic blood pressure per day after intravenous epinephrine pumping compared to no intravenous epinephrine).

**Figure 2.**
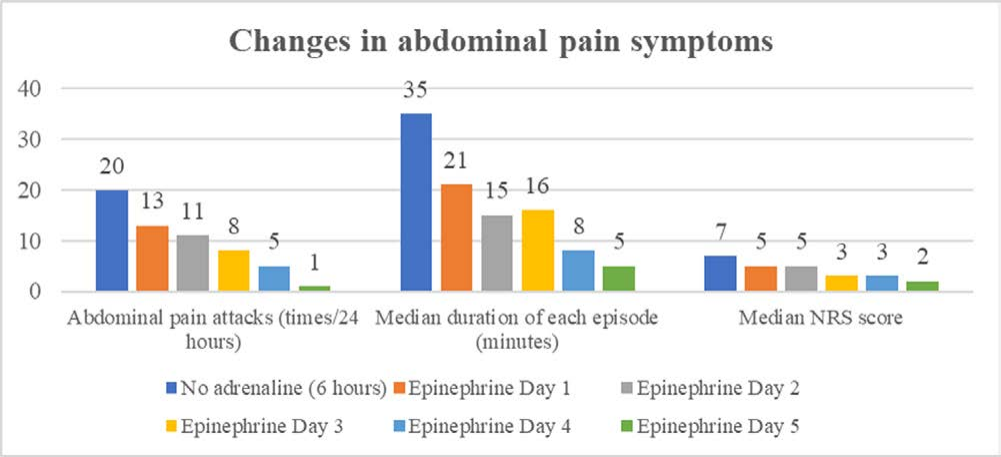
Changes in abdominal pain symptoms during intravenous epinephrine pumping.

During the treatment period, the patient had good compliance and no unexpected events occurred, and there was no statistically significant difference in his heart rate and blood pressure (Fig. 1), the frequency of abdominal pain attacks was significantly reduced, the duration of each attack was shortened, and the degree of pain gradually decreased. The patient’s abdominal pain symptoms improved significantly by March 5, with no recurrence (Fig. 2), and the rash and itching were reduced compared with before, the face and neck subsided, and the chest, back and limbs were still sporadic, and the continuous pumping of adrenaline was stopped. No abnormalities were found in the analysis of myocardial infarction markers. Continuous routine treatment continued until March 7th, and the patient’s skin rash improved and subsided. There were no further allergic symptoms as of March 8th (Table 1).

**Table 1:** Evolution of disease.

Time of disease progression	Clinical manifestation	NRS (maximum)	Therapy
6 hours before the present	Rash, abdominal pain, nausea, vomiting	5–6	Promethazine, loratadine
1 day	Rash, abdominal pain, nausea, vomiting	6–7	Methylprednisolone, promethazine, calcium gluconate, cetirizine, epinephrine
2 day	Rash, abdominal pain, nausea, vomiting	6–7	Methylprednisolone, diphenhydramine, calcium gluconate, cetirizine, loratadine, epinephrine
3 day	Rash, abdominal pain, nausea	5–6	Methylprednisolone, diphenhydramine, calcium gluconate, cetirizine, loratadine, epinephrine
4 day	Rash, abdominal pain	2–3	Methylprednisolone, diphenhydramine, calcium gluconate, cetirizine, loratadine, epinephrine
5 day	Rash, abdominal pain	0–1	Methylprednisolone, diphenhydramine, calcium gluconate, cetirizine, loratadine, epinephrine
6 day	Rash	0	Methylprednisolone, diphenhydramine, calcium gluconate, cetirizine, loratadine
7 day	Rash	0	Methylprednisolone, diphenhydramine, calcium gluconate, cetirizine, loratadine
8 day	–	0	Cetirizine

## 3. Discussion

Severe allergic reaction is a rapidly developing multisystem process that typically affects the skin, respiratory tract, gastrointestinal tract, and cardiovascular system. At present, there is still insufficient understanding of severe allergic reactions, especially in the progression of persistent allergic reactions to severe reactions. This case developed new gastrointestinal symptoms such as abdominal pain and vomiting on the 4th day of repeated rash occurrence, and the gastrointestinal symptoms persisted with paroxysmal exacerbation during subsequent treatment. The condition progressed to severe allergic reactions (grade I). Gastrointestinal urticaria is excluded based on the trigger of the allergy, the form of the rash, and the duration of the rash and gastrointestinal symptoms.

In the rescue of severe allergic reactions, adrenaline is the preferred drug for the treatment of grade II and above severe allergic reactions,^[3–6]^ and it is recommended to use it as early as possible after a clear diagnosis, and it is emphasized that intramuscular injection into the lateral thigh should be preferred.^[3,4,7]^ For patients with grade I reactions whose gastrointestinal symptoms are difficult to alleviate, intramuscular injection of adrenaline may also be considered.^[8,9]^ This patient only used antihistamines within the first 2 days of onset, and the symptoms were not controlled. Considering that glucocorticoids may reduce the risk of biphasic or delayed reactions,^[10,11]^ glucocorticoids were administered on the third day of onset, but the occurrence of persistent allergic reactions was not prevented, and the condition progressed to severe allergic reactions. After admission, glucocorticoids continued to be used, but skin, mucosal, and gastrointestinal symptoms persisted. Therefore, intramuscular injection of adrenaline was used according to the guidelines, with a dose ranging from 0.3 mg to 0.5 mg, and the rash and gastrointestinal symptoms were temporarily relieved within a short time after use.

However, the difficulty lies in the frequent recurrence of skin and gastrointestinal symptoms in patients after the end of adrenaline action. Based on the improvement of symptoms after intramuscular injection of adrenaline, changes in vital signs, and pharmacokinetics of adrenaline, we ultimately decided to break the conventional intramuscular injection method of adrenaline in the treatment of grade I severe allergic reactions. Under real-time electrocardiogram monitoring, a small dose of adrenaline was administered intravenously, maintaining a speed of 0.02 μg/kg/min, with a daily dose of approximately 2 mg, and intramuscular epinephrine is temporarily added when abdominal pain is unbearable during treatment. A total of 11.8 mg of epinephrine was administered from March 1 to 5, of which 2 successive intramuscular injections of 0.8 mg were given on March 1, followed by 0.5 mg of intramuscular epinephrine on the 2nd and 3rd days when abdominal pain was evident, and approximately 10 mg of adrenaline was continuously pumped intravenously during this period. During the whole period of epinephrine, the patient did not feel any new discomfort, there were no abnormal fluctuations in heart rate, heart rhythm and blood pressure, and the symptoms of rash and abdominal pain gradually improved.

Although the guidelines^[3]^ suggest that unnecessary intravenous administration should be avoided as much as possible to prevent adverse reactions effects from the use of adrenaline in drug treatment, we found through this case that for patients with persistent grade I severe allergic reactions, it is safe, feasible, and highly effective to use small doses of adrenaline intravenously under strict vital sign monitoring to prevent biphasic, persistent, or aggravated allergic reactions. Of course, the successful experience of one case is not extensive, but it is worth discussing and exchanging, and we will continue to collect such cases in the hope that it will promote the treatment of persistent severe allergic reactions in the future.

## Acknowledgments

The authors sincerely thank the family for giving permission to report this case.

## Author contributions

**Data curation:** Wei Wang.

**Resources:** Wei Wang, Ning Wang.

**Supervision:** Wen Hui Zhai, Li Tao, Tong-Xue Jiang.

**Writing – original draft:** Wei Wang, Ying Zhang, Ning Wang.

**Writing – review & editing:** Wei Wang, Tong-Xue Jiang.
